# Matching relations for optimal entanglement concentration and purification

**DOI:** 10.1038/srep25958

**Published:** 2016-05-18

**Authors:** Fan-Zhen Kong, Hui-Zhi Xia, Ming Yang, Qing Yang, Zhuo-Liang Cao

**Affiliations:** 1 School of Physics & Material Science, Anhui University, Hefei 230601, China; 2 Department of Computer Science, Jining University, Qufu, Shandong 273155, China; 3 Institute for Quantum Control and Quantum Information, School of Electronic and Information Engineering, Hefei Normal University, Hefei 230601, China

## Abstract

The bilateral controlled NOT (CNOT) operation plays a key role in standard entanglement purification process, but the CNOT operation may not be the optimal joint operation in the sense that the output entanglement is maximized. In this paper, the CNOT operations in both the Schmidt-projection based entanglement concentration and the entanglement purification schemes are replaced with a general joint unitary operation, and the optimal matching relations between the entangling power of the joint unitary operation and the non-maximal entangled channel are found for optimizing the entanglement in- crement or the output entanglement. The result is somewhat counter-intuitive for entanglement concentration. The output entanglement is maximized when the entangling power of the joint unitary operation and the quantum channel satisfy certain relation. There exist a variety of joint operations with non-maximal entangling power that can induce a maximal output entanglement, which will greatly broaden the set of the potential joint operations in entanglement concentration. In addition, the entanglement increment in purification process is maximized only by the joint unitary operations (including CNOT) with maximal entangling power.

Entanglement plays an important role in quantum information processing. It is a prerequisite in quantum teleportation^1^,^2^, entanglement swapping^3^, quantum cryptography^4^ and so on. Generating or enhancing entanglement between separated physical systems is of paramount importance in quantum information processing. Joint unitary operations can transform product states into maximal entangled states locally. In the nonlocal case (remote users), local operations can enhance the entanglement of non-maximally entangled states in a probabilistic way, such as entanglement concentration^5^ and entanglement purification^6^.

The idea of the standard entanglement concentration and purification processes is to extract a smaller number of more entangled pairs from a larger number of less entangled pairs by local operations and classical communications(LOCCs)^5^,^6^. The core part of the concentration and purification processes is the local CNOT operation on the two representatives from two different less entangled pairs. In experiment, the implementation of the CNOT operation is not a easy task, so efforts have been made to find easier ways to realize or to avoid the CNOT operation, such as in linear optical system^7^,^8^,^9^,^10^, in ionic or atomic system^11^,^12^ etc. In the purification of higher-dimensional entanglement, joint unitary operation also plays an very important role^13^,^14^,^15^,^16^. Pan *et al.* replaced the CNOT operation with a polarization beam splitter to purify polarization entangled mixed states^7^,^8^, and Gisin *et al.* replaced the CNOT operation with a general beam splitter to purify phase-error single-photon entangled states^9^,^10^. These results show that the complicated CNOT operation is not a necessity in entanglement concentration and purification, and the bilateral coupling between the two copies of the states to be concentrated or purified is the core part of an entanglement concentration or purification process. The role of this kind of coupling has been demonstrated^9^,^10^, and the optimal output fidelity is achieved with a general coupling rather than a CNOT-like coupling. But this result only applies to the phase-error single-photon entangled mixed states, and whether there exist the similar results for the purification of general mixed entangled states(such as Werner state) or the concentration of unknown non-maximally entangled pure states is not clear. In addition, the focus of entanglement concentration and purification is entanglement, so the entangling property of the coupling operation maybe more important than the coupling itself. So, in this paper, we will study the role of the joint unitary operation in a general entanglement concentration or purification process. Because it is not possible to get a pure maximally entangled state from purifying a finite ensemble of general mixed states, we will focus on the entanglement increment in purification process. Meanwhile, the maximal output entanglement is the most important target of entanglement concentration. Here, we are going to focus on the entangling power of the joint unitary operations at both sides, the parameter of the quantum channel and the entanglement of the output state (entanglement increment) so as to find the matching relation between the entanglement property of the joint unitary operations at both sides and the quantum channel to maximize the output entanglement (entanglement increment) in entanglement concentration (purification).

The entanglement property of a unitary operator has been studied from different aspects, such as entangling power^17^,^18^,^19^,^20^,^21^,^22^, entanglement measure^18^,^23^,^24^, entangling capacity^25^,^26^,^27^,^28^,^29^,^30^, and entanglement-changing power^31^. Entangling power of a joint unitary operation is defined as the mean entanglement (linear entropy) produced by applying the joint unitary operation on a given distribution of pure product states^17^. In this paper, we use the approach introduced in^17^ for measuring the entangling power of joint unitary operations, and concurrence is used to measure the entanglement of the output states^32^.

In this paper, we revisited the entanglement purification and the Schmidt-projection based entanglement concentration schemes with the CNOT operation being replaced with a general joint unitary operation, and the optimal matching relations between the entangling power of the joint unitary operation and the non-maximal entangled channel are found for optimizing the entanglement increment and output entanglement in entanglement purification and concentration.

## Results

### Matching relation in entanglement concentration

The bilateral CNOT operation in standard entanglement concentration process is replaced with a general bilateral bipartite operation, which is elaborated in the **Methods** section. The entanglement of the output state can be measured in terms of concurrence^32^. For the sake of brevity, the entanglement of the output state is called the output entanglement, and it is denoted by *C*_*out*_. The entanglement increment of the concentration process is denoted by Δ*C*. It is obvious that the joint control operation induced by the component *A*_*z*_ only does not have a positive contribution to the entanglement concentration. Hence, we only study the case in which 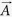 only has *A*_*x*_ or *A*_*y*_ component, and, for the sake of simplicity, the corresponding bilateral operations are referred to as controlled-*x* (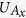) and controlled-*y* (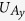) operations, respectively.

**Figure 1:**
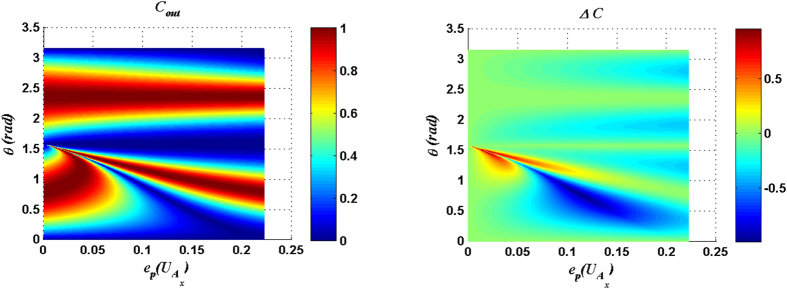
*C*_*out*_ and Δ*C* are plotted as functions of the parameter *θ* of input state and the entangling power of the joint unitary operation 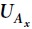. Here the measurement result is |00〉_34_.

**Figure 2:**
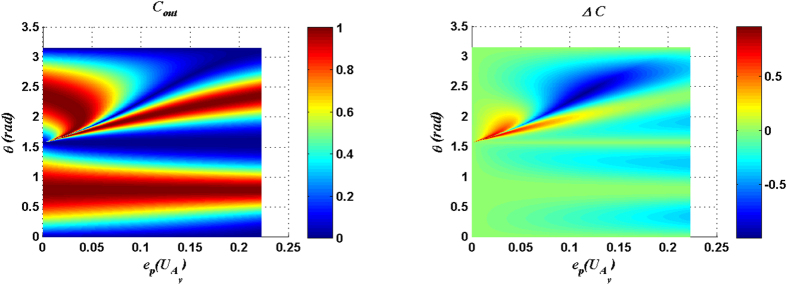
*C*_*out*_ and Δ*C* are plotted as functions of the parameter *θ* of input state and the entangling power of the joint unitary operation 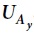. Here the measurement result is |00〉_34_.

For measurement result |00〉_34_, *C*_*out*_ and Δ*C* are plotted as functions of the parameter *θ* of input state and the entangling power of  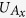 in Fig. 1, and *C*_*out*_ and Δ*C* are plotted in Fig. 2 for the case with 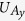.

Figure 1 (Fig. 2) shows that there exists an optimal matching relation between the entangling power of the 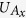 (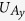) and the parameter *θ* of the input state so that *C*_*out*_ can reach the maximum value 1. The explicit expressions of the optimal matching relations for the cases with 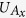 and 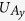 can be expressed respectively as:


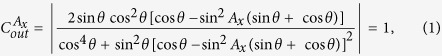



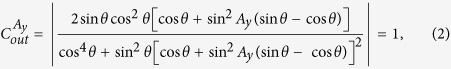


where 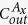 (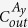) is the *C*_*out*_ for the case with 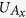 (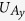).

Figures 1 and 2 also show that the considerable Δ*C* is presented around 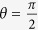 with a small entangling power of the joint unitary operation. From Figs 1 and 2, the following conclusion can be made: if the parameter *θ* of the input state is given a fixed value *θ*_0_ within the range 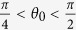 or 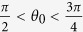, the solutions of Eq. (1) or (2) will fix some joint control operations which can not only produce the maximal *C*_*out*_ but also induce a considerable Δ*C*.

**Figure 3:**
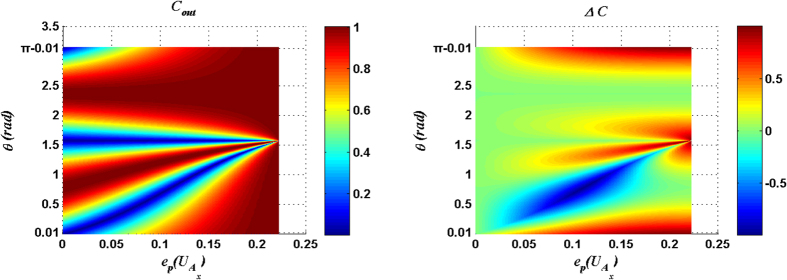
*C*_*out*_ and Δ*C* are plotted as functions of the parameter *θ* of input state and the entangling power of the joint unitary operation 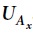. Here the measurement result is |11〉_34_.

**Figure 4:**
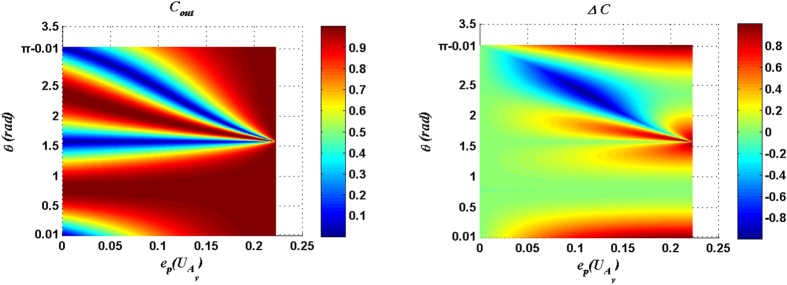
*C*_*out*_ and Δ*C* are plotted as functions of the parameter *θ* of input state and the entangling power of the joint unitary operation 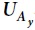. Here the measurement result is |11〉_34_.

Similarly, for the measurement result |11〉_34_, *C*_*out*_ and Δ*C* have been plotted as functions of the parameter *θ* of the input state and the entangling power of the joint operation 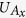 (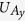) in Figs 3 and 4. Figs 3 and 4 show that, for the input states within the range 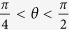 or 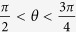, the optimal matching relation between the entangling power of the joint operation and the parameter *θ* of the input state always can be found for driving *C*_*out*_ to reach the maximum 1. Here the explicit expressions of the optimal matching relations can be expressed respectively as:


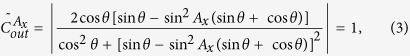



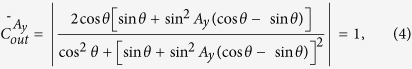


for the cases with 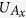 and 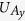. In addition, when the entangling power of the joint operation reaches the maximum value 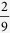, *C*_*out*_ is always the maximum 1 except for the product initial state case. This exception results from the fact that zero input entanglement leads to zero *C*_*out*_ in entanglement concentration. When the entangling power reaches the maximum value 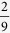, Δ*C* can be obtained as:


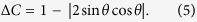


So when the input state is very close to a product state and the entangling power of the joint unitary operation reaches the maximum 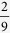, both *C*_*out*_ and Δ*C* are maximized for the measurement result |11〉_34_ case.

We can conclude that the set of joint unitary operations with maximal entangling power (including CNOT) is not the only set of joint operations which can maximize the *C*_*out*_ in entanglement concentration, and a variety of joint operations with lower entangling power can also induce a maximal *C*_*out*_, which will greatly broaden the set of the potential joint operations in entanglement concentration.

### Matching relation in entanglement purification

If the concentration process studied above is applied to mixed initial entangled states, it is called entanglement purification. Similarly, the joint control operation induced by the component *A*_*z*_ only does not have a positive contribution to the entanglement purification, so we only study the cases with 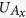 and 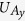, respectively.

**Figure 5:**
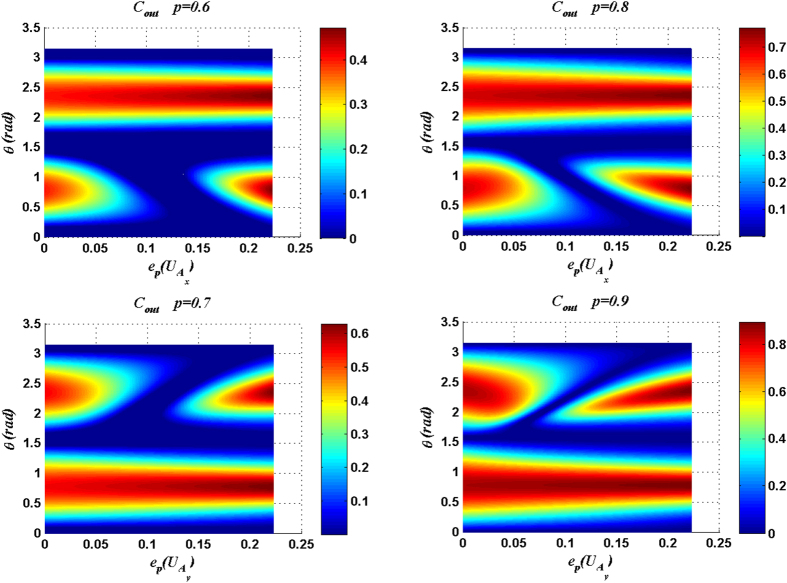
*C*_*out*_ (for different input purities *p*) are plotted as functions of the parameter *θ* of input state and the entangling power of the joint unitary operation 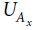 or 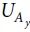. Here the measurement result is 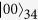.

**Figure 6:**
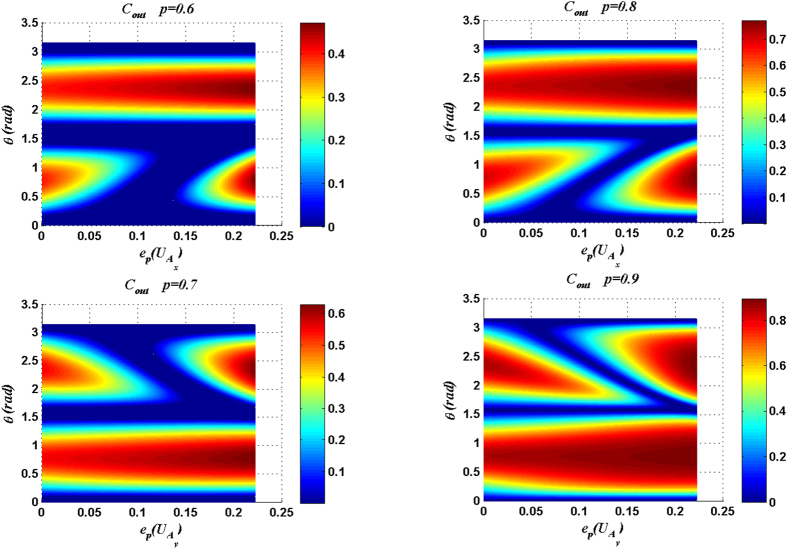
C_out_ (for different input purities *p*) are plotted as functions of the parameter *θ* of input state and the entangling power of the joint unitary operation 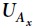 or 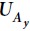. Here the measurement result is |11〉_34_.

Figures 5 and 6 show that the maximal *C*_*out*_ is increasing along with the purity *p* of the quantum channel. For a fixed purity of the quantum channel, the maximal *C*_*out*_ can be attained only when the entangling power of the joint unitary operation reaches the maximum 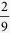 and the input entanglement reaches the maximum, i.e. the parameter *θ* is equal to 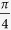 or 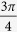. So, we can study the optimal matching relation between the channel and the entangling power of the joint operation for maximizing the Δ*C* by fixing 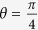. Because the output state for the measurement result |11〉_34_ case is a conjugate of that for the measurement result |00〉_34_ case when *θ* is equal to 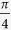, we only discuss the measurement result |00〉_34_ case.

**Figure 7:**
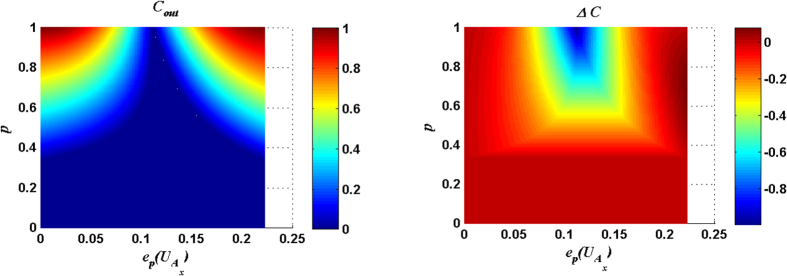
*C*_*out*_ and Δ*C* are plotted as functions of the purity *p* of the input state and the entangling power of the joint unitary operation 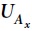. Here the measurement result is |00〉_34_, and 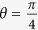.

**Figure 8:**
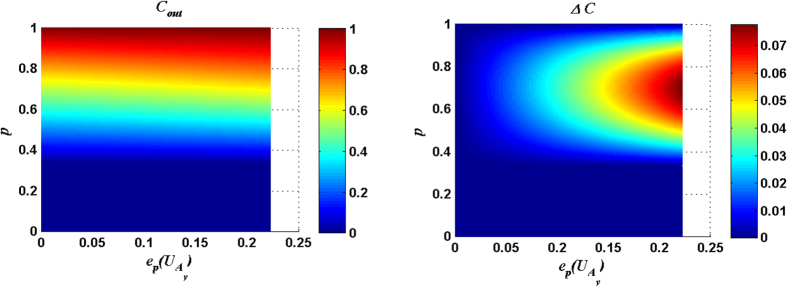
*C*_*out*_ and Δ*C* are plotted as functions of the purity *p* of the input state and the entangling power of the joint unitary operation 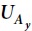. Here the measurement result is |00〉_34_, and 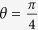.

Figures 7 and 8 show that for different initial purities *p* within the range 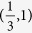, the maximal *C*_*out*_ can be achieved only when the entangling power of the joint unitary operation approaches the maximum value 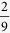. With the entangling power of the joint unitary operation being the maximum 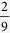 and the purity *p* of quantum channel being 0.6944, the only maximal Δ*C* (0.0775) can be reached. That is to say, the set of the joint unitary operations with the maximal entangling power (including CNOT) is required for achieving the maximal Δ*C* in entanglement purification. Although the CNOT operation in the standard entanglement purification is the optimal joint operation, our results still broaden the set of the potential joint operations in entanglement purification, because the CNOT operation is not the only joint operation whose entangling power is the maximum value 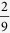.

## Conclusion

In this paper, by replacing the CNOT operation with a general joint operation, we studied the role of entangling power of the joint unitary operation in entanglement concentration and entanglement purification and found the matching relations between the entangling power of the joint unitary operation and the parameter of the quantum channel so that the entanglement of the output state or the entanglement increment is maximized. The results show that the set of joint unitary operations with maximal entangling power 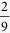 are needed for maximizing the entanglement increment of entanglement purification. But, for entanglement concentration, the result is somewhat counter-intuitive. Besides the set of joint unitary operations with maximal entangling power (including CNOT), there are a variety of joint operations with non-maximal entangling power that can induce a maximal output entanglement in entanglement concentration too, which will greatly broaden the set of the potential joint operations in entanglement concentration. In addition, because the CNOT operation is not the only joint operation whose entangling power is the maximum value 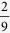, the CNOT operation in the standard entanglement purification is just a typical one of the optimal joint operations which can maximize the entanglement increment in entanglement purification. So the results presented here can greatly broaden the set of the potential joint unitary operations, which can maximize the output entanglement for entanglement concentration and the entanglement increment for entanglement purification, and may help the experimentalists to find a joint unitary operation which is both optimal and simple.

## Methods

### Entanglement concentration

Two pairs of particles (1, 2 and 3, 4) are prepared in non-maximally entangled pure states initially, and then distributed to two users Alice (1, 3) and Bob (2, 4). Each user will carry out a general control operation instead of CNOT operation on the two particles he (or she) possesses and then measure the target particle. If the target particles are in the same state, the source pair is retained. Next, we will analyze the relationship between the non-maximally entangled channel, entangling power of the general joint operation and the entanglement of the output state.

Suppose that particles (1, 2 and 3, 4) are in the following non-maximally entangled pure states:


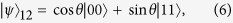



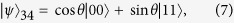


where 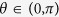. The bilateral control operation is in the following general form: 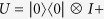

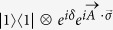
, where *e*^*iδ*^ is a relative phase factor, 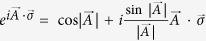, 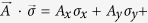

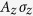
, and *σ*_*x*(*y*, *z*)_ is the Pauli operator. Here the first pair (1, 2) is the source pair, and the second pair (3, 4) is the target one. According to the definitions^17^,^20^, the entangling power of the general control operation *U* can be expressed as follows:


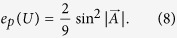


If the measurement after the control operation gives same results for both of the target particles 3, 4, then the source pair 1, 2 is retained and its state is regarded as the output state of the concentration process.

### Entanglement purification

If the concentration process studied above is applied to mixed initial entangled states, it is called entanglement purification. Without loss of generality, the initial mixed state to be purified can be expressed as the mixture of a pure non-maximally entangled state and an identity operator:


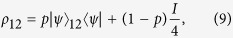



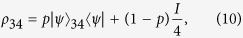


where 0 < *p* < 1, and 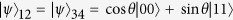. As discussed in the entanglement concentration process, if the target particles (3, 4) are measured in same states after the general joint controlled operation, the quantum state of the source particles 1, 2 is the output state, whose entanglement is calculated in terms of concurrence.

## Additional Information

**How to cite this article**: Kong, F.-Z. *et al.* Matching relations for optimal entanglement concentration and purification. *Sci. Rep.* **6**, 25958; doi: 10.1038/srep25958 (2016).

## References

[b1] Bennett C. H. *et al.* Teleporting an unknown quantum state via dual classical and Einstein-Podolsky-Rosen channels. Phys. Rev. Lett. 70, 1895 (1993). 10053414 10.1103/PhysRevLett.70.1895

[b2] Pirandola S., Eisert J., Weedbrook C., Furusawa A. & Braunstein S. L. Advances in quantum teleportation. Nature photonics 9, 641 (2015).

[b3] Zukowski M., Zeilinger A., Horne M. A. & Ekert A. K. “Event-ready-detectors” Bell experiment via entanglement swapping. Phys. Rev. Lett. 71, 4287 (1993). 10055208 10.1103/PhysRevLett.71.4287

[b4] Gisin N., Ribordy G., Tittel W. & Zbinden H. Quantum cryptography. Rev. Mod. Phys. 74, 145 (2002).

[b5] Bennett C. H., Bernstein H. J., Popescu S. & Schumacher B. Concentrating partial entanglement by local operations. Phys. Rev. A 53, 2046 (1996). 9913106 10.1103/physreva.53.2046

[b6] Bennett. C. H. *et al.* Purification of Noisy Entanglement and Faithful Teleportation via Noisy Channels. Phys. Rev. Lett. 76, 722 (1996). 10061534 10.1103/PhysRevLett.76.722

[b7] Pan J. W., Simon C., Brukner Č. & Zeilinger A. Entanglement purification for quantum communication. Nature 410, 1067 (2001). 11323664 10.1038/35074041

[b8] Pan J. W., Gasparoni S., Ursin R., Weihs G. & Zeilinger A. Experimental entanglement purification of arbitrary unknown states. Nature 423, 417 (2003). 12761543 10.1038/nature01623

[b9] Sangouard N., Simon C., Coudreau T. & Gisin N. Purification of single-photon entanglement with linear optics. Phys. Rev. A 78, 050301(R) (2008). 10.1103/PhysRevLett.104.180504 20482160

[b10] Salart D. *et al.* Purification of Single-Photon Entanglement. Phys. Rev. Lett. 104, 180504 (2010). 20482160 10.1103/PhysRevLett.104.180504

[b11] Yang M., Song W. & Cao Z. L. Entanglement purification for arbitrary unknown ionic states via linear optics. Phys. Rev. A 71, 012308 (2005).

[b12] Reichle R. *et al.* Experimental purification of two-atom entanglement. Nature 443, 838 (2006). 17051214 10.1038/nature05146

[b13] Horodecki M. & Horodecki P. Reduction criterion of separability and limits for a class of distillation protocols. Phys. Rev. A 59, 4206 (1999).

[b14] Alber G., Delgado A., Gisin N. & Jex I. Efficient bipartite quantum state purification in arbitrary dimensional Hilbert spaces. J. Phys. A 34, 8821 (2001).

[b15] Martín-Delgado M. A. & Navascués M. Distillation protocols for mixed states of multilevel qubits and the quantum renormalization group. Eur. Phys. J. D 27, 169 (2003).

[b16] Cheong Y. W., Lee S. W., Lee J. & Lee H. W. Entanglement purification for high-dimensional multipartite systems. Phys. Rev. A 76, 042314 (2007).

[b17] Zanardi P., Zalka C. & Faoro L. Entangling power of quantum evolutions. Phys. Rev. A 62, 030301 (2000).

[b18] Wang X. G., Sanders B. C. & Berry D. W. Entangling power and operator entanglement in qudit systems. Phys. Rev. A 67, 042323 (2003).

[b19] Clarisse L., Ghosh S., Severini S. & Sudbery A. Entangling power of permutations. Phys. Rev. A 72, 012314 (2005).

[b20] Ma Z. H. & Wang X. G. Matrix realignment and partial-transpose approach to entangling power of quantum evolutions. Phys. Rev. A 75, 014304 (2007).

[b21] Balakrishnan S. & Sankaranarayanan R. Entangling characterization of *SWAP*^1/*m*^ and controlled unitary gates. Phys. Rev. A 78, 052305 (2008).

[b22] Caruso F., Chin A. W., Datta A., Huelga S. F. & Plenio M. B. Entanglement and entangling power of the dynamics in light-harvesting complexes. Phys. Rev. A 81, 062346 (2010).

[b23] Zanardi P. Entanglement of quantum evolutions. Phys. Rev. A 63, 040304 (2001).

[b24] Balakrishnan S. & Sankaranarayanan R. Measures of operator entanglement of two-qubit gates. Phys. Rev. A 83, 062320 (2011).

[b25] Dür W., Vidal G., Cirac J. I., Linden N. & Popescu S. Entanglement Capabilities of Nonlocal Hamiltonians. Phys. Rev. Lett. 87, 137901 (2001). 11580626 10.1103/PhysRevLett.87.137901

[b26] Leifer M. S., Henderson L. & Linden N. Optimal entanglement generation from quantum operations. Phys. Rev. A 67, 012306 (2003).

[b27] Wang X. G. & Sanders B. C. Entanglement capability of a self-inverse Hamiltonian evolution. Phys. Rev. A 68, 014301 (2003).

[b28] Ye P. & Zheng Y. Z. Entanglement capabilities of non-local Hamiltonians with maximally entangled ancillary particles. Phys. Lett. A 328, 284 (2004).

[b29] Chefles A. Entangling capacity and distinguishability of two-qubit unitary operators. Phys. Rev. A 72, 042332 (2005).

[b30] Campbell E. T. Optimal entangling capacity of dynamical processes. Phys. Rev. A 82, 042314 (2010).

[b31] Ye M. Y., Sun D., Zhang Y. S. & Guo G. C. Entanglement-changing power of two-qubit unitary operations. Phys. Rev. A 70, 022326 (2004).

[b32] Wootters W. K. Entanglement of Formation of an Arbitrary State of Two Qubits. Phys. Rev. Lett. 80, 2245 (1998).

